# Natural Law in the Business World

**Published:** 1887-06

**Authors:** 


**Natural Law in the Business World.** By HENRY WOOD. Boston: Lee & Shepard. New York: C. T. Dillingham. Price, 75 cts.

This book is well worth the reading. The light of natural law is applied to the live, social and economic topics which are now attracting so much attention. It aims to expose the abuses and evils which masquerade under the banner of Labor, and the bad results of class prejudice and antagonism. Labor combinations, and their effect on the laborer; socialistic tendencies; excess of economic and railroad legislation; the distribution of wealth; principles governing corporations and railroads, and also many other prominent issues, are fully and thoroughly examined, in their connection with unvarying natural laws and principles. It is shown clearly that the business world is permeated by natural law, and that success in any department can only be gained by conformity to it. The opposing combinations, unions, corners, unwarranted legislation, sentimental and socialistic ideas, and everything else of an artificial nature, are shown to be mischievous, destructive and on a false basis. Every one who has read Drummond's "Natural Law in the Spiritual World," and many more, will be interested in seeing a corresponding application of natural and fixed principles to the economic and business world in which we live.

